# Menopause Is Associated with Obstructive Sleep Apnea in a Population-Based Sample from Mecklenburg–Western Pomerania, Germany

**DOI:** 10.3390/jcm12062101

**Published:** 2023-03-07

**Authors:** Markus Krüger, Anne Obst, Till Ittermann, Olaf Bernhardt, Tatyana Ivanovska, Marek Zygmunt, Ralf Ewert, Ingo Fietze, Thomas Penzel, Reiner Biffar, Amro Daboul

**Affiliations:** 1 Poliklinik für Zahnärztliche Prothetik, Alterszahnmedizin und Medizinische Werkstoffkunde, Universitätsmedizin Greifswald, 17475 Greifswald, Germany; 2 Klinik und Poliklinik für Innere Medizin B, Universitätsmedizin Greifswald, 17475 Greifswald, Germany; 3 Institute for Community Medicine, University Medicine Greifswald, 17475 Greifswald, Germany; 4 Department of Restorative Dentistry, Periodontology, Endodontology, Preventive Dentistry and Pediatric Dentistry, University Medicine Greifswald, 17475 Greifswald, Germany; 5 Fakultät für Elektrotechnik, Medien und Informatik, Ostbayerische Technische Hochschule Amberg-Weiden, 92224 Amberg, Germany; 6 Department of Obstetrics and Gynecology, University of Greifswald, 17489 Greifswald, Germany; 7 Interdisziplinäres Schlafmedizinisches Zentrum, Charité–Universitätsmedizin Berlin, 10117 Berlin, Germany

**Keywords:** obstructive sleep apnea, obstructive sleep apnea syndrome, sleep apnea, apnea-hypopnea index, sleep-disordered breathing, body mass index, BMI, AHI, OSA, ordinal regression, splines, study of health in Pomerania (SHIP), menopause, menstrual cycle, polysomnography

## Abstract

*Objective:* Menopause is associated with multiple health risks. In several studies, a higher incidence or a higher risk for obstructive sleep apnea (OSA) in post-menopausal than pre-menopausal women is reported. This study was designed to verify such a connection between menopause and OSA in a population-based sample. *Methods:* For a subsample (N = 1209) of the Study of Health in Pomerania (N = 4420), complete polysomnography data was available. Of these, 559 females completed a structured interview about their menstrual cycle. Splines and ordinal regression analysis were used to analyze the resulting data. *Results:* In the ordinal regression analysis, a significant association between the apnea–hypopnea index (AHI) and menopause indicated that post-menopausal women had a substantially higher risk of OSA. In accordance with previous studies, risk indicators such as body mass index (BMI), age, and the influence of hysterectomies or total oophorectomies were included in the model. *Conclusions:* Our results clearly confirmed the assumed connection between menopause and OSA. This is important because OSA is most often associated with male patients, and it warrants further research into the underlying mechanisms.

## 1. Introduction

Obstructive sleep apnea (OSA) is a sleep disorder that is associated with the repetitive collapse of the upper airway during sleep, which results in fragmented sleep, recurrent arousals, and intermittent periods of hypoxia and hypercapnia [1]. It has been receiving increasing attention in the past decade, largely because of the growing research linking OSA to cardiovascular disease, metabolic disorders, and a decline in neurocognitive and behavioral functions [2,3].

Although the pathophysiology of the disorder is not well established and a multifactorial origin is suggested, previous epidemiological studies pointed out that OSA is associated with aging, the male gender, craniofacial anatomy, and obesity [4].

There is mounting evidence for post-menopausal women having a higher risk of developing OSA, and at the same time—women are at risk of being underdiagnosed for this particular health risk [5] (for an overview, see Shaver and Zenk [6] and Perger, Mattalano, and Lombardi [7]).

### 1.1. Empirical Findings

There are at least two population-based studies in which participants are asked about their menstrual cycle to determine their menopausal status and in which the AHI was measured by an overnight polysomnography (PSQ). For a population sample (N = 535) from Sao Paulo, Brazil, Hachul et al. [8] reported a higher AHI in post-menopausal women than in pre-menopausal ones. This is in line with data from the Wisconsin Sleep Cohort Study at two different measuring points (N = 589, N = 219) [9,10]. In all these studies, results were adjusted for known factors of relevance, such as age and BMI.

### 1.2. Possible Mechanisms

The mechanism that links menopause to OSA is still not clear. In some studies [11,12], the disruptions in the female reproductive hormones are considered as a possible factor, whereby a hormonal imbalance during menopause and post-menopause can alter the distribution of body fat and increase fat accumulation in the trunk and abdomen area [13,14]. Nonetheless, it remains unclear if this increase in fat accumulation is independent of age and/or obesity.

In a recent review on OSA and menopause, a different mechanism was anticipated by which reductions in estrogen and/or progesterone hormones might cause instability in the ventilatory control system, leading to an overall increased risk for OSA and a degraded response to apneic events [15]. Both hysterectomy and oophorectomy are associated with a higher OSA risk [16].

### 1.3. The Apnea–Hypopnea Index

The AHI is not an ideal measure for diagnosis or the severity grading of OSA; however, so far, no better measure or combination of measures has been generally accepted [17,18]. In the present study, we limit our interpretation to higher AHIs on a group level as an indication of a higher risk of being afflicted by OSA or developing OSA on an individual level.

### 1.4. Aims and Scopes

As there is a strong indication for a connection between menopause and OSA, the aim of our current study was to verify such an effect in a large population-based sample from Pomerania, Germany, while taking into account relevant variables such as BMI and age as well as potential confounders (e.g., whether hormone influencing organs have been surgically removed).

## 2. Methods

### 2.1. Sample

The sample was a part of the Study of Health in Pomerania (SHIP) [19,20]. SHIP is a population-based cohort study monitoring the health status in Pomerania, Germany. Participants of the first measuring point of the cohort SHIP-Trend, were offered to visit a sleep laboratory for an overnight polysomnography [21]. Of the 4420 participants of the SHIP-TREND-0 cohort, 1264 accepted the offer. Complete AHI data was available for 1209 (559 [46%] females) persons.

### 2.2. Polysomnography

An overnight attended Polysomnography (PSG) was conducted according to the standards of the American Academy of Sleep Medicine (AASM) using ALICE 5 PSG devices (Philips Respironics, Eindhoven, Netherlands). All sensors were carefully placed by trained and certified staff [22]. All PSG recordings were digitally stored and transferred to the University Hospital Charité, Center of Sleep Medicine Berlin, Germany, where sleep stages of 30-s epochs and respiratory events were evaluated visually by certified sleep technicians according to AASM 2007 criteria. (Note: For Hypopnea scoring, a desaturation of at least 4% and airflow drop of at least 30% were used (A criteria]. [23,24]) The AHI was calculated as the number of apnea/hypopneas per hour of sleep estimated by the total sleep time. The severity of sleep apnea was defined according to AHI: No sleep apnea—AHI < 5 per hour sleep, Mild sleep apnea—AHI 5–15 per hour sleep, Moderate sleep apnea—AHI 15–30 per hour sleep, Severe sleep apnea—AHI ≥ 30 per hour sleep. (Further details on the PSG setup and recordings can be found in Appendix A).

### 2.3. Data Analysis

For data analysis and visualization, R Statistics and splines [25] with the packages haven [26], dplyr [27], tidyr [28], MASS [29], rstatix [30], and ggplot2 [31] were required.

### 2.4. Risk Indicators

Only females were included in the ordinal regression analysis. Descriptive data of the whole PSG sample and possible biases are readily available elsewhere [21,24,32,33].

Age in years (*M* = 53 years, SD = 13, range 20–81) was computed from the participants’ date-of-birth on the day of the examination.

For the BMI (*M* = 28.2, SD = 5.5, range: 18.4–50.1), participants were measured and weighed in their underwear.

For the indicator Post-Menopause, participants were asked if they have had their menopause yet (no = 197, yes = 362) and, if true, at what age their last menstruation took place. Two participants who could or would not answer that question were counted as “no”.

For the indicator Uterus, participants were asked whether their uterus had been removed (yes = 99, no = 460). One participant who could or would not answer that question was counted as “no”.

As for the indicator Ovaries, participants were asked if both of their ovaries were removed (yes = 34, no = 525). One participant who could or would not answer that question was counted as “no”.

### 2.5. Outcome

The AHI (*M* = 7.3, SD = 11.3, range: 0–82.4) was measured and computed as described in the paragraph *Polysomnography* (see also Appendix B).

## 3. Results

### 3.1. Descriptive Data

**Figure 1:**
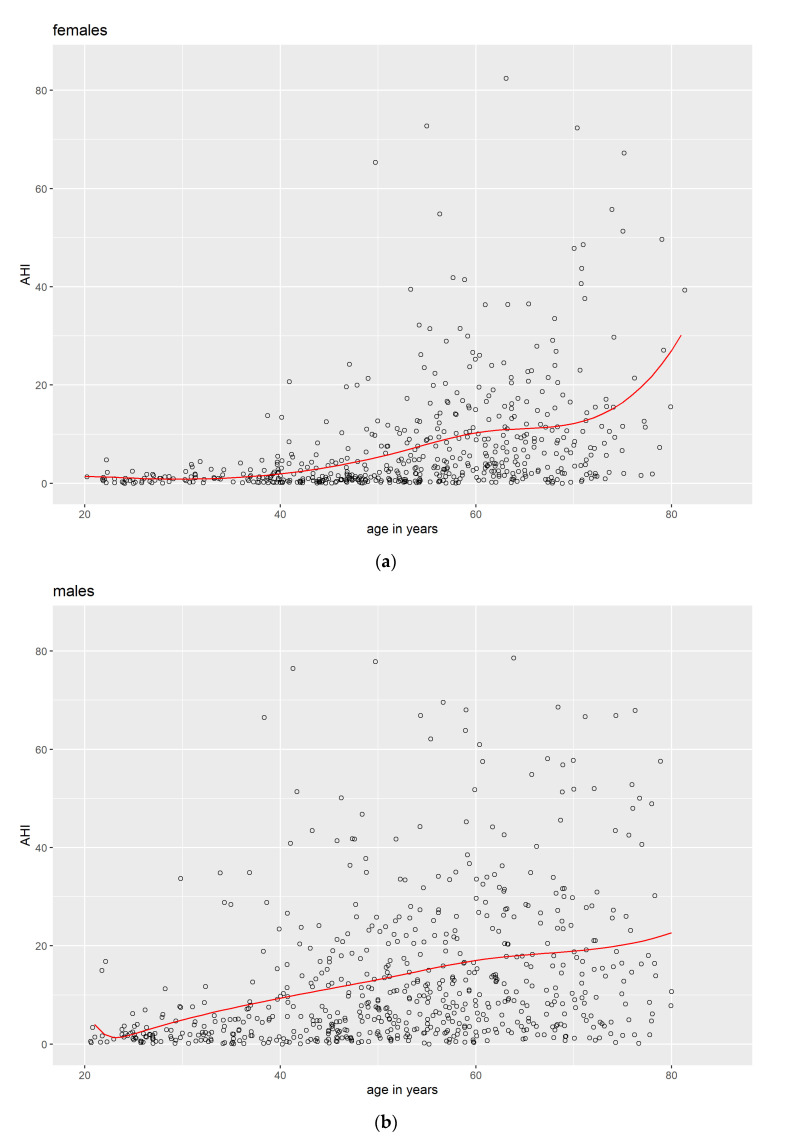
Scattered data points (black circles) and splines (red line) with knots at 25, 52, and 61 years for age and AHI separately for (**a**) females and (**b**) males.

**Figure 2:**
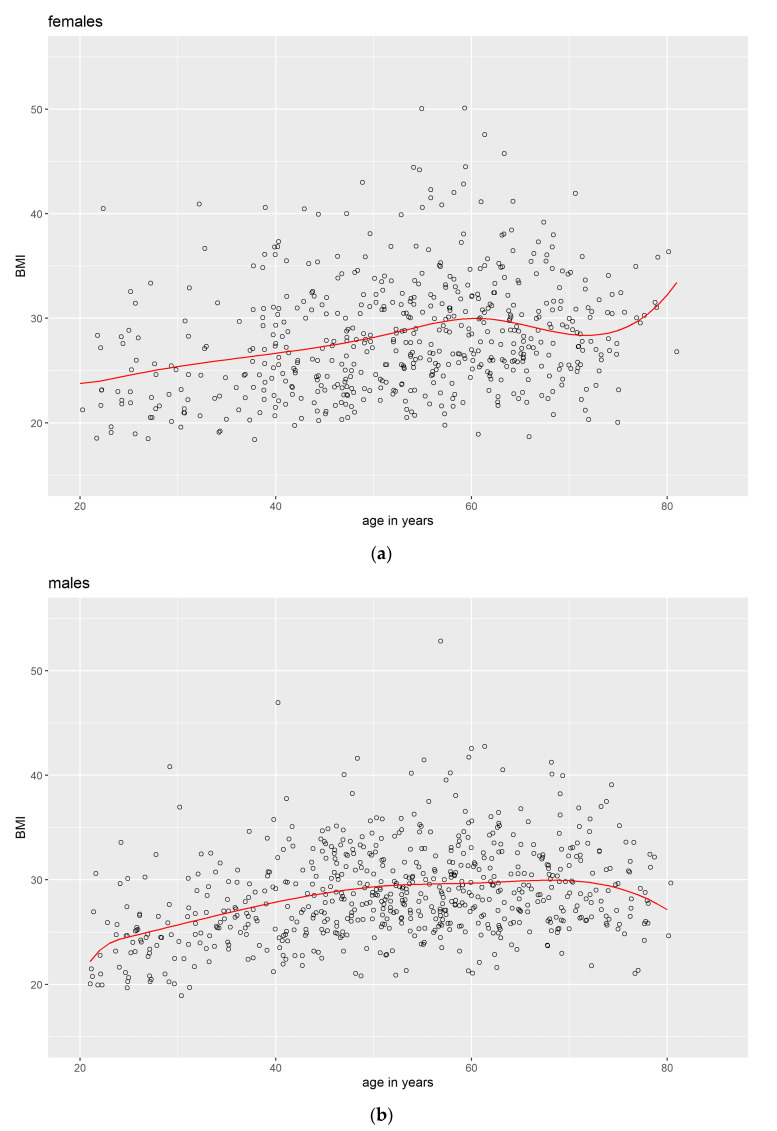
Scattered data points (black circles) and splines (red line) with knots at 25, 52, and 61 years for age and BMI separately for (**a**) females and (**b**) males.

For a better understanding of the data, cubic unrestricted splines [34] were computed for age and AHI (Figure 1), as well as for age and BMI (Figure 2) separately for males and females. Knots for the splines were chosen at the lowest, the median, and the highest age women reported their transition to menopause—25 years, 52 years, and 61 years. For determining the knots, only female participants with a uterus and at least one ovary were included (see Methods).

A visual inspection of the graphs indicated a rather different course for the AHI over age for females compared with males. In males, we observe an almost linear trend with a rising AHI over the years, whereas in females, there is a rise in the AHI with age, too, but it is rather unsteady. It seems that—on a group level—after a long plateau phase, at about 40 years, the AHI rises slowly from a harmless level to an AHI above 10, which is associated with mild sleep apnea. This rise seems to come to an end at about 60+ years, shortly after the last women reported their transition to menopause. This might very well be associated with menopause. The last stretch of the females’ graph should not be interpreted because there were very few participants around the age of 80 years. For the course of BMI over age, no differences can be seen between males and females that can easily be connected to menopause.

Descriptively, the mean AHI of post-menopausal women (*M* = 10.3, SD = 12.9) was higher than that of pre-menopausal women (*M* = 1.9, SD = 3.3), Cohen’s *d* = 0.89 (for any analyzes of significance, see Section 3.2 below).

### 3.2. Ordinal Logistic Regression Analysis

An ordinal logistic regression with multiple continuous indicators (Age (in years) and BMI (kg/m²)) and multiple dichotomous indicators (Post-Menopause (no, yes), Uterus (not removed, removed), and Ovaries (at least one intact ovary, both ovaries removed)) and on AHI severity (AHI < 5: normal, 5 ≤ AHI < 15: mild, 15 ≤ AHI < 30: moderate, AHI ≥ 30: severe) was computed.

Significant effects were found for the predictors: Age, *p* < 0.001, BMI, *p* < 0.001, and Post-Menopause, *p* = 0.005 and all others, *p* > 0.10. With all other introduced indicators held constant, post-menopausal women had a 182% higher chance (OR 2.82, [CI 1.38, 5.94]. Note: Odds ratios in ordinal regressions are indicating the chance of being in the next higher category of the categorical target variable. This is in contrast to the logistic regression, where the target variable is dichotomous.) of a higher severity grade of sleep apnea as indicated by the AHI. (A multiple linear regression with the AHI as a continuous variable mirrored the results of the ordinal logistic regression with a significant effect for the indicator Menopause, too, with all variance inflation factors below 10.)

## 4. Discussion

As expected, age and BMI were significant risk indicators for AHI in women. This was also true for the indicator Menopause, indicating that menopause is an important factor in our sample from Pomerania, Germany—even beyond the well-established risk indicators of age and BMI. Looking at the women’s splines for age and AHI (Figure 1a), it becomes obvious that the timeframe when menopause takes place is associated with a notable increase in the AHI. This increase subsides shortly after the last women reached menopause. Apparently, this is not reflected in the women’s splines for age and BMI (Figure 2a).

This later finding is in line with findings that post-menopausal women experience reduced musculature and build up fat without any obvious changes in their weight [35]. Accordingly, the higher AHI in post-menopausal women would depend on the fat distribution and not on an overall change in body weight [5,14] (in contrast to a higher AHI in post-menopausal women being mediated by a general rise in body weight that is in turn, a consequence of the menopause [36,37]). In future research, one could utilize the proportion of body fat as an indicator instead of the BMI.

Certainly, there are other sleep disorders (e.g., insomnia, restless legs syndrome) connected with menopause (for an overview, see Eichling and Sahni [36]). Interactions with OSA might occur [38]. Often, poor sleep in post-menopausal women is associated with depression [33,39]. In a subsample of the Wisconsin Sleep Cohort Study, Young et al. [40] found that sleep quality problems reported by post-menopausal women are not easily verifiable with a PSG. Taking into account various components of sleep architecture, it seemed that the post-menopausal women had better sleep quality than the pre-menopausal ones. Nevertheless, any signs of mood disorders or sleep disorders must be taken seriously. In the light of the present findings, the cumulative evidence for the risk of OSA in post-menopausal women and the considerable long-term cardiovascular risks associated with OSA [2,3,41], it seems reasonable to screen patients for OSA when trouble sleeping or daytime sleepiness is reported.

### 4.1. Strength and Limitations

SHIP [19,20] is a long-running study with meticulous quality control. All measures are checked for plausibility, reliability, and validity constantly. Our participants had undergone a gold standard PSG and were questioned about their menstrual cycle in a standardized interview. This guarantees high data quality.

Despite a planned and stratified recruitment for SHIP, self-selection bias by the participants is hard to disprove. In our present study, such an effect might be elevated as the PSG was not required for participation in SHIP, but was offered as an extension. Participants might have declined due to time constraints that are systematically inherent to subsamples (e.g., working persons or persons involved in childcare might be less likely to find the time for an overnight PSG) [42]. Furthermore, we assume that persons are more likely to participate when they are unsatisfied with their sleep (e.g., in our sample, persons aware of their snoring are more likely to choose the PSG [32]).

While the cross-sectional design of our study limits the interpretation of causality, it seems more plausible that menopause and the underlying hormonal changes cause the observed higher AHI and not the other way round.

A lack of significant effects for the factors Uterus and Ovaries are to be considered carefully. In the case of ordinal regressions, one cannot draw the conclusion that statistically non-significant predictors are evidence for no effect.

### 4.2. Conclusions and Perspectives

In our current study, we were able to replicate the findings of two other population-based studies confirming a higher OSA risk in post-menopausal women. We expanded these findings by visualizing this effect using splines. While our study cannot contribute to disentangling the (most likely) hormonal mechanism causing this effect, it strongly underlines the need for continued research, by providing further evidence for the often-detrimental influence of menopause on women’s health.

## Data Availability

The data that support the findings of this study are available from the Transferstelle für Daten- und Biomaterialienmanagement (Office for transfer of data and bio materials) of the University Medicine Greifswald, Study of Health in Pomerania (SHIP: https://www.fvcm.med.uni-greifswald.de/dd_service/data_use_intro.php (accessed on 1 November 2022)). Access is restricted and needs the approval of the board.

## Abbreviations

AASM	American Academy of Sleep Medicine
AHI	Apnea–hypopnea index
ANOVA	Analysis of variance
BMI	Body mass index
CI	Confidence interval
DFG	Deutsche Forschungsgemeinschaft
EEG	Electroencephalogram
ECG	Electrocardiogram
EOG	Electrooculogram
OSA	Obstructive sleep apnea
PSQ	Polysomnography
SD	Standard deviation
SHIP	Study of Health in Pomerania

## Appendix A

Overnight-attended PSG was conducted according to the standards of the American Academy of Sleep Medicine (AASM) using ALICE 5 PSG devices (Philips Respironics, Eindhoven, Netherlands). All sensors were carefully placed by trained and certified staff. Recordings included six channels of electroencephalogram (EEG, F4-M1, C4-M1, O2-M1, F3-M2, C3-M2, O1-M2), two channels of electrooculogram (EOG), two channels of electromyogram (chin and tibialis muscles), one electrocardiogram (ECG), inductive plethysmography detecting thoracic and abdominal movements, nasal pressure sensor, pulse oximetry, a microphone to detect snoring, and a body position sensor. Bio-calibration was performed before the light was switched off (eye movement, teeth grinding, breathing, snoring, and leg movement). All PSG recordings were digitally stored and transferred to the University Hospital Charité, Center of Sleep Medicine Berlin, Germany, where sleep stages of 30-s epochs and respiratory events were evaluated visually by certified sleep technicians according to AASM 2007 criteria. The apnea–hypopnea index was calculated as the number of apneas/hypopneas per hour of sleep estimated by the total sleep time. The severity of sleep apnea was defined according to the apnea–hypopnea index (AHI): No sleep apnea—AHI < 5 per hour sleep, Mild sleep apnea—AHI 5–15 per hour sleep, Moderate sleep apnea—AHI 15–30 per hour sleep, Severe sleep apnea—AHI ≥ 30 per hour sleep.

## Appendix B

**Table A1:** Number of participants with no (AHI < 5), mild (5 ≤ AHI < 15), moderate (15 ≤ AHI < 30), or severe (AHI ≥ 30) sleep apnea according to the AHI and the corresponding mean BMI (SD) of these subgroups separate for the pre- and post-menopausal individuals.

	No OSA	Mild OSA	Moderate OSA	Severe OSA	Total
**Pre-menopausal**	N = 180	N = 14	N = 3	N = 0	N = 197
	BMI = 26.0 (4.9)	BMI = 32.5 (3.9)	BMI = 37.7 (4.6)		BMI = 26.7 (5.3)
**Post-menopausal**	N = 170	N = 110	N = 57	N = 25	N = 362
	BMI = 27.0 (4.3)	BMI = 29.8 (5.2)	BMI = 32.7 (5.8)	BMI = 32.0 (6.0)	BMI = 29.1 (5.4)
**Total**	N = 350	N = 124	N = 60	N = 25	
	BMI = 26.5 (4.6)	BMI = 30.1 (5.1)	BMI = 33.0 (5.8)	BMI = 32.0 (6.0)	
